# FTIR Spectroscopy for Evaluation and Monitoring of Lipid Extraction Efficiency for Oleaginous Fungi

**DOI:** 10.1371/journal.pone.0170611

**Published:** 2017-01-24

**Authors:** Kristin Forfang, Boris Zimmermann, Gergely Kosa, Achim Kohler, Volha Shapaval

**Affiliations:** 1 Department of Mathematical Sciences and Technology, Norwegian University of Life Sciences, Ås, Norway; 2 Nofima AS, Ås, Norway; Agency for Science Technology and Research, SINGAPORE

## Abstract

To assess whether Fourier Transform Infrared (FTIR) spectroscopy could be used to evaluate and monitor lipid extraction processes, the extraction methods of Folch, Bligh and Lewis were used. Biomass of the oleaginous fungi *Mucor circinelloides* and *Mortierella alpina* were employed as lipid-rich material for the lipid extraction. The presence of lipids was determined by recording infrared spectra of all components in the lipid extraction procedure, such as the biomass before and after extraction, the water and extract phases. Infrared spectra revealed the incomplete extraction after all three extraction methods applied to *M*.*circinelloides* and it was shown that mechanical disruption using bead beating and HCl treatment were necessary to complete the extraction in this species. FTIR spectroscopy was used to identify components, such as polyphosphates, that may have negatively affected the extraction process and resulted in differences in extraction efficiency between *M*.*circinelloides* and *M*.*alpina*. Residual lipids could not be detected in the infrared spectra of *M*.*alpina* biomass after extraction using the Folch and Lewis methods, indicating their complete lipid extraction in this species. Bligh extraction underestimated the fatty acid content of both *M*.*circinelloides* and *M*.*alpina* biomass and an increase in the initial solvent-to-sample ratio (from 3:1 to 20:1) was needed to achieve complete extraction and a lipid-free IR spectrum. In accordance with previous studies, the gravimetric lipid yield was shown to overestimate the potential of the SCO producers and FAME quantification in GC-FID was found to be the best-suited method for lipid quantification. We conclude that FTIR spectroscopy can serve as a tool for evaluating the lipid extraction efficiency, in addition to identifying components that may affect lipid extraction processes.

## Introduction

The growing global demand of lipids as a source of food, feed and fuel has caused an increasing interest in microbial production of lipids. Numerous microalgae, yeasts, filamentous fungi and some bacteria are referred to as oleaginous microorganisms due to their ability to accumulate over 20% (w/w) lipids [1–4]. Microbial lipids, often referred to as single cell oils (SCOs), mainly consist of fatty acids in the form of triglycerides. Depending on the fatty acid composition, the SCO can be exploited in the production of nutraceuticals, food, feed or biodiesel. Knowledge of the fatty acid yield and profile of the SCO are crucial for determining the potential application. The first step in determining the yield and fatty acid profile is lipid extraction. While a wide variety of lipid extraction methods are in use, it is known that variations in experimental conditions and solvent polarities between the different methods result in a high variability in the reported fatty acid yields and profiles from similar SCO producers [5–7].

Lipids are normally extracted in the laboratory with chemical methods using organic solvents [8–10]. For lipid extraction from SCO producers, there is often a need to combine solvent extraction with physical, biological or other chemical methods to break down cell surface structures [11]. Physical methods include pressing and mechanical cell disruptions, using expeller presses, bead beating, ultra-sonication, microwaves or electroporation [12–16]. Biological methods are based on degradation of cell surface structures using specific enzymes [17] while chemical methods include acid or base hydrolysis or more recently explored solvents to permeabilize the cell wall [18–20]. In the process of selecting a suitable SCO producer, a large number of samples should be assessed. Common lab-scale extraction is carried out by using a mixture of chloroform and methanol, which forms a monophasic solution when it is mixed with the endogenous water of the sample. This chloroform-methanol method was first developed [21] and modified by Folch, Lees (8), and later improved by Bligh and Dyer (9). Both methods are widely cited and considered as standards in the field of lipid extraction. Nevertheless, numerous modifications have been introduced through the years, some being presented as modifications [22–24] while others are still being referred to as the original methods [25, 26]. Another standard lab-scale extraction is the Soxhlet method [10, 27], in which the solvent system, usually hexane, passes through the sample after evaporation and condensation in a semi-continuous fashion. Although this method works well for large samples, it is tedious and not applicable to very small sample sizes [28]. Lately, more attention has been given to direct or *in situ* transesterification methods [29–32]. In these methods, extraction and transesterification are combined in a single step, applied directly to the microbial biomass. The fatty acid content of the SCO is converted into fatty acid methyl esters (FAMEs) that can be analyzed by gas chromatography (GC). The direct transesterification methods allow for higher sample throughput compared to conventional extraction methods by omitting the step between extraction and transesterification [33–36]. One of these methods, the Lewis direct transesterification [33], is a method widely employed in SCO extraction from oleaginous microalgae but remains to our knowledge not evaluated for extraction from oleaginous fungi.

Since the efficiency of a lipid extraction method strongly depends on the type of microbial biomass the method is applied to, the extraction efficiency needs to be evaluated for each individual case. This is commonly done by comparing the performance of an extraction method with standard methods such as Bligh, Folch or Soxhlet. However, such a comparison can only reveal the relative extraction efficiency and does not indicate whether residual lipids remains in the biomass after extraction using any of the methods in the comparison. Thus, an analytical tool that allows monitoring residual lipids in the biomass would be very valuable. While the amount of lipid residuals may be roughly estimated by sequential extractions from the biomass [35, 37], this is often avoided since it is laborious and time-consuming. Therefore, there is a need for a rapid and reliable tool that can estimate the residual lipid content of the biomass and the efficiency of the extraction process simultaneously.

Fourier Transform Infrared (FTIR) spectroscopy is an analytical tool that is widely used for the identification and characterization of microorganisms [38–44]. FTIR spectroscopy can be directly applied to microorganisms in suspensions. It allows fast and economical detection of the main components of biological material (lipids, proteins, nucleic acids and carbohydrates) by their specific absorbance frequencies. Previous studies have shown that FTIR spectroscopy can be used to determine lipid accumulation in oleaginous microorganisms [45, 46] and to identify the main fatty acids [47, 48]. Although FTIR spectroscopy does not perform a direct quantitative fatty acid analysis, it represents an excellent tool for monitoring the *in situ* lipid content of biomass.

The goal of the present work was to introduce FTIR spectroscopy as a tool for monitoring lipid extraction efficiency in fungal biomass by evaluating different lipid extraction methods for the oleaginous fungi *Mucor circinelloides* and *Mortierella alpina*. The studied species, *Mucor circinelloides* and *Mortierella alpina*, are considered as models for oleaginous microorganisms and have been important in the industrial production of γ-linolenic acid (GLA) and arachidonic acid (ARA), respectively [49, 50]. Firstly, FTIR spectroscopy was used to monitor the extraction efficiency of the Bligh, Folch and Lewis direct transesterification methods. Secondly, FTIR spectroscopy was used to evaluate two biomass pretreatment approaches, bead beating and acid hydrolysis, for increased extraction efficiency for the investigated strains. Finally, the gravimetric lipid yield and the GC-derived FAME content were compared for different lipid quantification techniques after lipid extraction. The study revealed that FTIR spectroscopy has enormous potential in the optimization and monitoring of extraction processes applied to biological material. Simultaneously, the technique can be used to identify components that can affect lipid extraction processes and result in differences in extraction efficiency.

## Materials and Methods

### Chemicals, Microorganisms and Cultivation Conditions

All solvents applied in this study were of GC-grade. The oleaginous fungi *Mucor circinelloides* VI04473 and *Mortierella alpina* UBOCC-A-112046 were obtained from the Norwegian Veterinary Institute (Oslo, Norway) and the University of Brest Culture Collection (Brest, France), respectively. Fungal strains were cultured in 200 mL N-limiting medium containing 80 gL^-1^ glucose and 3 gL^-1^ yeast extract at 28°C with 120 rpm shaking. More information about the cultivation conditions are given in supporting information (S1 Text). Fungal biomass was harvested by vacuum-filtration after 120 hours of cultivation, washed three times with distilled water and lyophilized at -52°C and 0.0010 mbar for 24h (Christ, Alpha 1–2 LD plus, Germany).

### Extraction Pretreatment

Acid hydrolysis was carried out by suspending the biomass (50 and 35 mg of *M*.*circinelloides* and *M*.*alpina*, respectively) in 2mL 3N HCl and incubating the sample at 80°C for 1 h. The acid was diluted by the addition of water and removed. The biomass sample was lyophilized prior to extraction for removal of residual water and used directly for lipid extraction. Bead beating was carried out by mixing 35–50 mg biomass with 250–300 mg beads (710–840 μm, Merck, Germany) and 1 mL distilled water in a microtube and homogenizing the sample with a Fastprep®-24 Instrument set to 4.0 m/s for 60 sec. Bead beating exposures of 1, 3 and 5 minutes were tested. Residual water was removed from the sample by lyophilization and all material was used directly for lipid extraction.

### Lipid Extraction and Transesterification

Lyophilized biomass, 50±2 and 35±1 mg of *M*.*circinelloides* and *M*.*alpina*, respectively, was weighted into a 14 mL glass centrifuge tube for extraction. Different extraction methods were carried out as described below.

#### Folch extraction

The method of Folch, Lees (8) was modified in order to meet the requirements of the small sample size (1g = 1mL). The sample was first soaked in distilled water to create a water content of 80%_w_ and then homogenized in chloroform/methanol (2:1, v/v) using a glass rod. The solvent/sample ratio was 20:1. The solvent phase was separated from the biomass by centrifugation (2800 g, 10 min) and subsequently transferred into a new centrifuge tube. The solvent phase was then washed with 0.2x solvent volume with 0.88% KCl and centrifuged to separate the water and extract phases. The extract was collected in a pre-weighted glass tube and the solvent was evaporated under a steam of nitrogen at 60°C. Lipids were transesterified using the Lewis direct transesterification method and analyzed by GC-FID.

#### Bligh & Dyer Extraction

As for the Folch extraction, the method of Bligh and Dyer (9), referred to as the Bligh method, was adapted to meet the requirements of the small sample size (1g = 1mL). The sample was soaked in distilled water (see Folch extraction) and homogenized in methanol/chloroform (2:1, v/v) using a glass rod. The initial solvent/sample ratio was 3:1 and the sample was further homogenized by adding additional chloroform, reaching a final solvent/sample ratio of (3+1):1. The solvent phase was separated from the biomass by centrifugation (2800 g, 10 min), transferred to a new tube and washed with distilled water to create a final chloroform/methanol/water ratio of 2:2:1,8. The extract was transferred to a pre-weighted tube and the solvent was evaporated at 60°C under a steam of nitrogen. Lipids were transesterified using the Lewis direct transesterification method and analyzed by GC-FID.

#### Lewis direct transesterification

The method described by Lewis, Nichols (33) was used for the direct transesterification of fungal biomass and as transesterification method prior to GC analysis of the Folch and Bligh extracts. Biomass or dried extracts were dissolved in 2 mL methanol/chloroform/HCl (10:1:1, v/v). Biomass was homogenized with a glass rod. The internal standards, tridecanoic acid (C13:0, Sigma Aldrich, USA) and tricosanoic acid (C23:0, Sigma Aldrich, USA) were dissolved in hexane and added to a final amount of 1.0 and 0.7 mg per sample, respectively. Another 1 mL of methanol/chloroform/HCl (10:1:1, v/v) was added to the extraction tube. The sample was vortexed for 30 sec and incubated at 90°C for 1 hour. Samples were cooled down and 1mL water was added. FAMEs were extracted with 2 x 2mL hexane/chloroform (4:1, v/v) after phase separation by centrifugation (2800 g, 10min). The extract was collected in a pre-weighted tube and the solvent was evaporated under a steam of nitrogen. FAMEs were dissolved in 1 mL hexane with 0.01% butylated hydroxytoluene (BHT, Sigma-Aldrich, USA) and subsequently transferred into a GC vial, which was stored at -20°C until analysis.

### TLC Analysis

The solvent phase was dried under nitrogen gas at 60°C and lipids were dissolved in 100 μl of hexane (final amount of lipids ~15 mg). Free fatty acids (FFAs), phospholipid (PL), monoacylglycerols and diacylglycerols (MDGs) and TAGs were separated by thin-layer chromatography (TLC) on a silica gel plate (Sigma Aldrich, USA) using a mixture of petroleum ether, diethyl ether and acetic acid (113:20:2 *v*/*v*/v) as the mobile phase. Lipids were visualized by dipping the plates in copper sulfate solution. The spots corresponding to FFAs, PLs, MDGs and TAGs were identified by comparison with known standards by a Bioscan AR-2000 Radio-TLC & Imaging Scanner (Bioscan Inc., Washington, DC, USA). Quantification was performed by scraping off each individual band and dissolving it in hexane. The hexane mixture was vortexed and centrifuged (2800 rpm for 10 min) to remove the silica gel, the hexane phase was dried under nitrogen gas at 60°C and lipids were quantified gravimetrically.

### FAME Analysis by GC-FID

Gas chromatography was performed on a Hewlet Packard 6890 instrument equipped with a SGE BPx70 column, 60 m length, 0.25 mm ID and 0.25 μm film thickness. Helium was used as the carrier gas at a flow rate of 1.4 mL/min. One microliter of the sample was injected in 20:1 split mode. The inlet and detector temperatures were 280°C. The oven temperature was initially set to 70°C for 1 min, followed by an increase of 30°C/min to 170°C, an increase by 1.5°C/min to 200°C, and finally an increase by 3°C/min to 220°C and hold for 5 min. The post run temperature was set to 70°C. FAMEs were identified by comparison with GLC-85 and GLC-463 standard mixtures of FAMEs (NU-CHEK-PREP, Inc., USA).

### Quantification of Lipids

Quantification of lipids was carried out by three different approaches in order to evaluate variability between techniques for quantification of lipids and FAMEs. (1) Gravimetric approach: lipids were quantified gravimetrically by evaporating the solvent under a steam of nitrogen and determining the weight of the lipid extract. (2) Internal standard approach: the internal standards, C13:0 and C23:0 added during the transesterification step, were used to quantify the FAME content in GC-FID. An average response factor was calculated (added IS amount/integrated area of IS) for each sample and used to quantify all fatty acids in the sample by multiplying area and response factor. (3) External standard calibration: an 18919-1AMP FAME mix (Supelco, USA) was prepared as a working solution of 10 mg/mL and run in a dilution series of 1:1, 1:2, 1:4, 1:6, and 1:10 in GC-FID. Response factors (RF) were determined for all components (C_n_) in the mixture based on corresponding calibration curves and were used to quantify the FAME content of the samples by the following equation:

$$Normalized\;FAME\;yield=\frac{Area\;C_{n}\;\times RF\;C_{n}}{DW\;biomass}\;\times \;\frac{Amount\;C_{13:0\;added}}{Amount\;C_{13:0\;measured}}$$

### FTIR Analysis

For FTIR measurements, a High Throughput Screening eX-Tension (HTS-XT) unit coupled to a Vertex 70 spectrometer (both Bruker Optik GmbH, Germany) equipped with a globular mid-IR source and a DTGS detector were used. Intact fungal biomass (before extraction, BE) and residual biomass (after extraction, AE) were transferred to microtubes and dissolved in 0.5 mL distilled water. The sample was sonicated by a 2mm probe coupled to a VC 505 ultrasonic processor (Sonics & Materials, USA) for 2 x 30 sec under 40% amplitude power. Following the sonication, three aliquots, each containing 10 μL of the biomass suspension, were transferred onto IR-light transparent silicon plates with 384 wells (Bruker Optik GmbH, Germany). The samples were dried in a desiccator with silica gel (Merck, Germany) to generate a thin film for IR measurements. Samples of the extract (EP) and water (WP) phases were applied several times and dried in between to circumvent problems related to the surface tension of solvents. A total amount of 10 μL was needed to reach an optimal film thickness for FTIR transmission measurements of the EP and WP samples. Spectra were recorded in transmission mode in the spectral region 4000 to 500 cm^-1^ with a resolution of 6 cm^-1^, taking 64 scans for both background and sample spectra. Prior to each sample well FTIR measurement, background spectra were collected by measuring an empty well on the silicon plate. The presented FTIR spectra are representative spectra of two replicate experiments of the same treatment were three spectra were collected per treatment and an average spectrum was calculated. All spectra were normalized by multiplicative signal correction (MSC) in the environment of Unscrambler X 10.3.

## Results and Discussion

### Evaluating the Efficiency of Bligh, Folch and Lewis Extraction Methods by FTIR Spectroscopy

**Fig 1:**
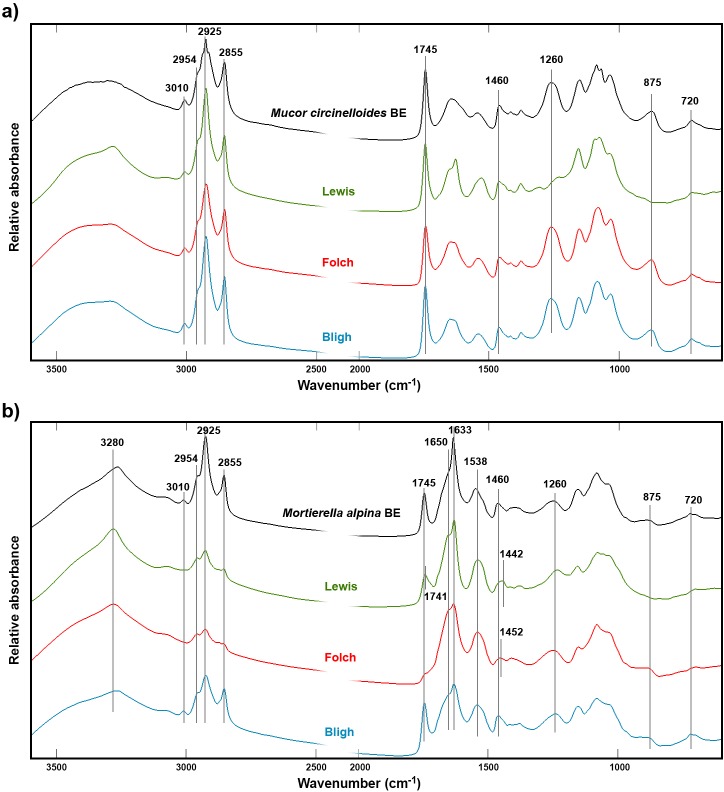
Infrared spectra of fungal biomass before and after lipid extraction. FTIR spectra recorded of *Mucor circinelloides* (A) and *Mortierella alpina* (B) biomass before (BE) and after extraction of lipids using the methods of Bligh, Folch and Lewis.

**Fig 2:**
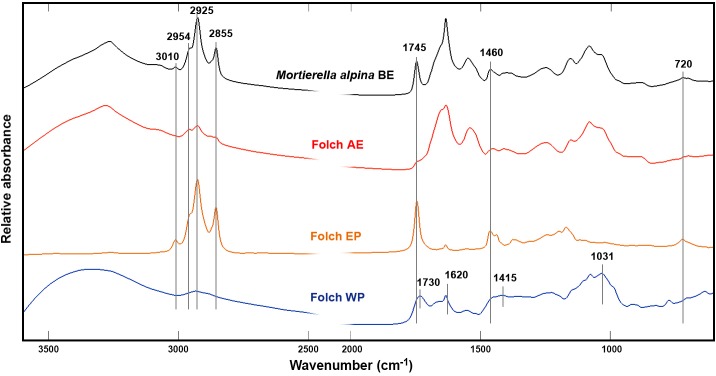
Infrared spectra of all extraction phases after Folch extraction. Lipids were extracted from *Mortierella alpina* biomass using the Folch method and infrared spectra were recorded of the biomass before (BE) and after extraction (AE), the extract phase (EP) and the water phase (WP).

The Bligh, Folch and Lewis extraction methods were applied in order to extract lipids from *Mucor circinelloides* and *Mortierella alpina* biomass. In order to evaluate the efficiency of the three extraction methods for *M*.*circinelloides* and *M*.*alpina*, FTIR spectra of the biomass were recorded before (BE) and after (AE) extraction (Fig 1), as well as of the water (WP) and extract (EP) phases (Fig 2). Fig 1 clearly shows that the lipid content of the biomass after extraction is considerable for *M*.*circinelloides* (Fig 1A), while for *M*.*alpina* (Fig 1B) lipids were only present in the biomass after extraction by the Bligh method.

The lipid content of the fungal biomass, can be identified by peaks related to C-H stretching vibrations (= C-H stretch at 3010 cm^-1^; C-H stretching in–CH_3_ and CH_2_ at 2855, 2925 and 2954 cm^-1^), C = O stretching in esters (1745 cm^-1^), CH_2_ bending (1460 cm^-1^), C-O-C stretching in esters (1070–1250 cm^-1^) and CH_2_ rocking (720 cm^-1^) [51, 52]. All these principal signals of lipids are clearly visible in the spectrum of extracted lipids shown in Fig 2. Corresponding lipid signals were not detected in the spectra of the water phases after extraction. However, signals of glucuronic acid (1730, 1620 and 1100–900 cm^-1^) and signals related to glycerol (2933–2835, 1415, and 1031 cm^-1^) could be detected in the water phase (WP) spectrum after Folch extraction and transesterification, respectively (Fig 2). These compounds were identified by comparing the spectrum to the FTIR spectra of glucuronic acid and glycerol (Fig A in S1 Fig).

After extraction of lipids from *M*.*alpina* biomass, the FTIR spectra of the residual biomass showed lipid signals when the Bligh method was applied, while when the Lewis or Folch methods were used no lipid signals could be observed (Fig 1B).

The IR spectrum of *M*.*alpina* biomass after Folch extraction (Fig 1B) did not show absorbance peaks related to unsaturated hydrocarbons, C = O stretch and CH_2_ rocking at wavenumbers 3010, 1745 and 720 cm^-1^, respectively. Bands resulting from CH stretching vibrations at 2855, 2925 and 2954 cm^-1^ are still visible. This is explained by the fact that biological material exhibits CH stretching vibrations due to the presence of–CH_3_ and–CH_2_- in cellular components like proteins and carbohydrates in addition to lipids. This can be seen in the biomass spectra of *M*.*alpina* and *M*.*circinelloides* grown under non-lipid accumulating conditions (no nitrogen limitation), which are given in Fig B of S1 Fig. However, the considerable decrease in intensity of the CH signals (2855, 2925 and 2954 cm^-1^) in the spectrum of *M*.*alpina* biomass after extraction indicates a successful extraction of lipids using the Folch method. Further, a shift of maxima from 1460 to 1452 cm^-1^ of the CH_2_ bending signal was observed. We hypothesize that the shifted bands are not associated with lipids but with CH_2_ bending signals in either proteins or chitin, a major component (present as chitin-glucan complexes) in the fungal cell wall [53, 54]. This is supported by the co-existence of additional peaks observed in relation to NH stretching (3280 cm^-1^), C = O stretching (amide I: 1650, 1633 cm^-1^) and C-N-H vibration (amide II: 1538 cm^-1^) [55].

The spectrum of *M*.*alpina* biomass after Lewis extraction (Fig 1B) displayed a high C = O stretching peak that was shifted to 1741 cm^-1^. Other lipid peaks were low or absent, thus indicating a fat-free biomass: The absence of absorbance peaks at 720 and 3010 cm^-1^, the decreasing intensities at 2855, 2925 and 2954 cm^-1^ and the shifts of peaks at 1460 and 1745 cm^-1^, all indicate that the IR spectrum of *M*.*alpina* after Lewis extraction is fat-free. This is supported by the calculated FAME yield for the different extraction methods (Table 1, no pretreatment). Lipid contents of *M*.*alpina* grown on glucose have been reported in the range 12.5–57.1% [23, 56–61]. Therefore, lipid yields determined in our study of 29.0% and 21.3% for Lewis and Folch extractions, respectively, are in accordance with these previous findings. For the spectrum of *M*.*alpina* biomass after Lewis extraction, peaks appeared at 1741 and 1442 cm^-1^, and were shifted compared to the original biomass where the corresponding bands appeared at 1745 and 1460 cm^-1^, respectively. These shifts may again be associated with the cell wall components chitin and glucuronans. Due to the use of hydrochloric acid (HCl) in the Lewis extraction method, hydrolysis of the ester bond present in the N-acetyl-β-D-glucosamine units of chitin may be induced, which converts the chitin units into chitosan units (D-glucosamine). This reduces the degree of acetylation in chitin and can be responsible for the shift of peaks related to NH and C = O stretching in the infrared spectrum. Moreover, hydrochloric acid-induced hydrolysis of glucuronans can shift C = O stretching signals of glucuronic acid units that are typically at 1720–1740 cm^-1^ [62, 63].

**Table 1:** Lipid extraction yield after fungal biomass extraction using the methods of Bligh, Folch and Lewis and different biomass pretreatments.

FAME yield (mg/g biomass)				
Strain	Extraction method	No pretreatment	Acid hydrolysis pretreatment	Bead beating pretreatment
*M*. *circinelloides*	Bligh	128.3 ± 34.8	210.8 ± 22.5	104.5 ± 13.7
	Folch	113.0 ± 13.2	283.0 ± 84.1	253.3 ± 17.8
	Lewis	258.4 ± 3.8	269.7 ± 34.9	313.5 ± 17.5
*M*. *alpina*	Bligh	6.8 ± 3.8	104.4 ± 16.2	122.4 ± 11.6
	Folch	212.6 ± 66.3	163.7 ± 0.9	221.3 ± 44.4
	Lewis	289.8 ± 2.9	273.8 ± 17.6	334.0 ± 8.7

While Bligh extraction resulted in lower lipid yield for both fungal species, an extremely low yield of 6.8 mg/g biomass was obtained for the Bligh extraction for *M*.*alpina* (Table 1). It has been demonstrated by Iverson, Lang [64], in marine samples with lipid contents exceeding 2%, that the low solvent-to-sample ratio of the Bligh method can underestimate the lipid yield. However, this cannot explain the extremely low yield after Bligh extraction in *M*.*alpina*. We hypothesize, that the low lipid yield in *M*.*alpina* biomass is rather explained by the difficulty of separating the lyophilized *M*.*alpina* biomass (without pretreatment) from the solvent causing solvent residuals to be left in the extraction tube. The actual yield should be closer to 100–120 mg/g, which is still much lower than the yield obtained for Folch extraction from *M*.*alpina* biomass (Table 1). To show that the observed underestimation was caused by a low solvent-to-sample ratio, we increased the initial solvent/sample ratio of the Bligh method from 3:1 to 20:1 (similar to Folch method). We observed a significant increase in lipid yield from 6.7 to 285.2 mg/g biomass in *M*.*alpina*, which was similar to the yield after Folch extraction (212.6 mg/g). The FTIR spectra of the biomass before (BE) and after extraction (AE) using the different solvent/sample ratios in the Bligh method are given in the supporting information (Fig B in S1 Fig). The biomass spectrum after extraction with the modified Bligh method showed that peaks at 720, 1745 and 3010 cm^-1^ are absent, the intensities is drastically decreasing at 2855, 2925 and 2954 cm^-1^ and that the peak at 1460 is shifted. Therefore, we consider the biomass to be lipid free after extraction using a modified Bligh method.

### Differences in SCO Extraction Efficiency between *M*.*circinelloides* and *M*.*alpina*

In the present study, significant differences were observed in the extraction efficiency between *M*.*circinelloides* and *M*.*alpina*. A higher lipid yield (21.2%) after Folch extraction in *M*.*alpina* indicates that lipids are more easily extracted from this species compared to *M*.*circinelloides* (11.3%) (Table 1, no pretreatment). The lipid content of *M*.*circinelloides* is typically around 25% [65], although gravimetric lipid yields up to 44% also have been reported [66]. The FTIR spectrum of the *M*.*alpina* biomass after Folch extraction was fat-free, while the spectrum of *M*.*circinelloides* after Folch extraction showed presence of residual lipids (Fig 1). This difference in extraction efficiency may be explained by differences in the cell wall structure between these two species. The IR spectrum of *M*.*circinelloides* biomass before extraction (BE) showed high intensities at 875 and 1260 cm^-1^ related to P-O and P = O stretching which could be explained by the presence of polyphosphates in the cell wall of *Mucor* species [67–69] (Fig 1A). These peaks disappear in the spectrum of the biomass after Lewis extraction, indicating that the HCl employed during extraction hydrolyzes the polyphosphates located between the cell wall and the cytoplasmic membrane. The removal of polyphosphates from the biomass could facilitate the extraction of lipids by increasing the permeability of the cell wall to organic solvents. Low intensities of peaks associated with P = O stretching (875 and 1260 cm^-1^) in the IR spectrum of the *M*.*alpina* biomass before extraction (BE, Fig 1B) indicate a lower concentration of polyphosphates in this species. This may also explain the more efficient lipid extraction from *M*.*alpina* biomass compared to *M*.*circinelloides* using the Folch method, since this method does not include acid hydrolysis. To our knowledge, this is the first report of the possible negative effects of cell wall polyphosphates on the lipid extraction from fungal biomass. This feature clearly demonstrates how FTIR spectroscopy can be used not only for monitoring but also for understanding the lipid extraction process.

### Effect of Biomass Pretreatments

**Fig 3:**
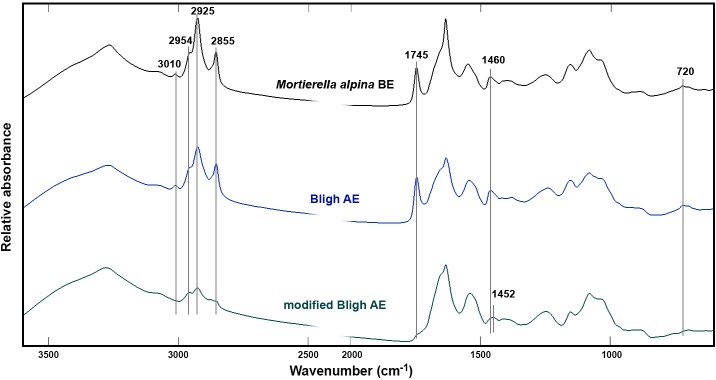
Infrared spectra of *M*.*circinelloides* biomass after acid hydrolysis pretreatment and lipid extraction. *Mucor circinelloides* biomass was subjected to acid hydrolysis and infrared spectra of the biomass were recorded before (BE) and after pretreatment and extraction of lipids using the methods of Bligh, Folch and Lewis.

In order to increase the efficiency of lipid extraction from the filamentous fungus *M*.*circinelloides*, we pretreated the biomass before extraction. We tested two biomass pretreatment methods, acid hydrolysis and bead beating, and subsequently performed the Lewis, Folch and Bligh extraction methods for *M*.*circinelloides* and *M*.*alpina* biomass. The FAME yields for different combinations of pretreatments and extraction methods showed that the extraction efficiency in both *M*.*circinelloides* and *M*.*alpina* could be increased for almost all extraction methods by applying acid hydrolysis or bead beating before lipid extraction (Table 1), except when acid hydrolysis was combined with Folch extraction for *M*.*alpina* and when bead beating was combined with Bligh extraction for *M*.*circinelloides*. For the acid hydrolysis combined with Folch extraction in *M*.*alpina*, the lower FAME yield might be related to the loss of lipids associated with the removal of acid from the biomass before extraction. The large margin of error reported for the Bligh extraction without pretreatment could explain why bead beating does not seem to increase the FAME yield for Bligh extraction from *M*.*circinelloides* biomass. Both the FAME yields (Table 1) and the infrared spectra of *M*.*circinelloides* biomass after extraction (Fig 3) indicate that the acid hydrolysis was slightly more effective for Folch extraction compared to Lewis extraction.

**Fig 4:**
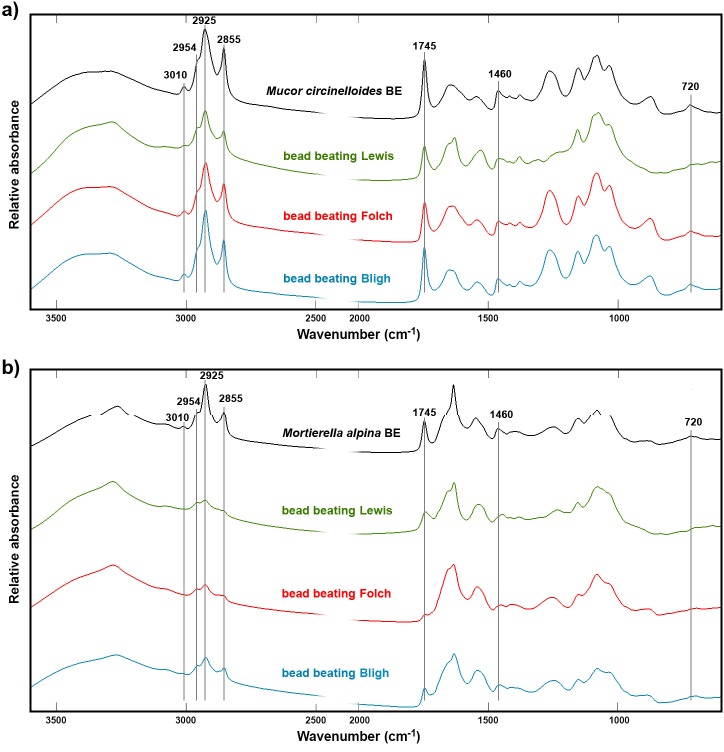
Infrared spectra of fungal biomass after bead beating pretreatment and lipid extraction. *Mucor circinelloides* (A) and *Mortierella alpina* (B) biomass was subjected to bead beating pretreatment and infrared spectra were recorded of the biomass before (BE) and after extraction of lipids using the methods of Bligh, Folch and Lewis. Bead beating exposure time was 1 min.

The intensities in the range 3010–2855 cm^-1^ and at 1460 cm^-1^ of the biomass spectra were lowerafter Folch extraction compared to the spectrum after Lewis extraction. The FTIR spectra of *M*.*alpina* biomass after acid hydrolysis and lipid extraction are given in Fig D in S1 Fig. The effect of bead beating pretreatment in the two fungal species is shown in Fig 4.

The IR spectra of *M*.*circinelloides* biomass after bead beating combined with Folch extraction (Fig 4B) still showed presence of fat, as can be observed by absorption signals related to lipids (3010, 2855, 1925, 2954, 1745, 1460, 1170–1250 and 720 cm^-1^). Contrarily, bead beating combined with Folch extraction from *M*.*alpina* biomass (Fig 4B) showed that lipids were extracted with a high efficiency. The results indicate that acid needs to be included for efficient Folch extraction of lipids from *M*.*circinelloides* biomass. The use of acid can either be integrated in the extraction method, as for the Lewis direct transesterification, or it can be part of a pretreatment step. This provides additional proofs of the previously mentioned negative effects of cell wall polyphosphates on lipid extraction in *M*.*circinelloides*.

Inclusion of bead beating as a pretreatment slightly increased the FAME yield for Lewis extraction in both fungal species (Table 1). However, the IR spectrum of *M*.*circinelloides* biomass after extraction and bead beating (Fig 4A) revealed that part of the lipids remained in the biomass. Therefore, we tested whether an increase in bead beating time could increase the efficiency of the extraction methods in *M*.*circinelloides* (Fig E in S1 Fig). An increase in bead beating time did not significantly improve the FAME yield for Bligh and Folch extractions, but a clear improvement was observed when the pretreatment time was increased from 1 min to 5 min for the Lewis method, resulting in an increase in FAME yield from 313.5 to 623.4 mg/g biomass (Table A in S1 Table). The same effect was visible in the FTIR spectra of *M*.*circinelloides* biomass after Lewis extraction, in which the intensities related to lipid content (3010, 2855, 2925, 2954, 1745, 1460, 1170–1250 and 720 cm^-1^) decreased in conjugation with increasing pretreatment exposure (Fig E in S1 Fig). From the infrared spectra, we can deduce that at least 5 minutes bead beating (under similar conditions) should be employed in the Lewis method when extracting fatty acids from *M*.*circinelloides*. This effect was not so evident for the Bligh and Folch extractions combined with bead beating at different exposure times (Fig E in S1 Fig). Therefore, mechanical disruption of the biomass was shown to be the most effective biomass pretreatment process when acid hydrolysis or an extraction method employing acid (e.g. Lewis) were utilized.

### Lipid Quantification

The yield of lipids extracted from *M*.*circinelloides* and *M*.*alpina* biomass using the different extraction methods were determined by three different approaches: (1) gravimetric approach, (2) internal standard approach, and (3) external standard calibration. A high variability in lipid yields was observed when the different extraction methods and quantification techniques were applied (Table 2).

**Table 2:** Quantification of lipids in fungal biomass using gravimetric, internal standard and external calibration approaches.

Lipid yield (mg/g biomass)				
Strain	Extraction method	Gravimetric yield^a^	IS quantification yield^b^	External calibration yield^c^
*M*. *circinelloides*	Bligh	162.2 ± 2.54	138.3 ± 26.9	128.3 ± 34.8
	Folch	237.7 ± 5.2	115.0 ± 12.8	113.0 ± 13.2
	Lewis	447.3 ± 31.1	273.8 ± 1.1	258.4 ± 3.8
*M*. *alpina*	Bligh	84.2 ± 1.0	7.3 ± 3.7	6.8 ± 3.7
	Folch	436.1 ± 43.7	233.9 ± 67.8	212.6 ± 66.3
	Lewis	486.2 ± 68.5	301.0 ± 3.0	289.8 ± 2.9

^a.^ Gravimetric weight after evaporation of solvents.

^b.^ Conversion of lipids into FAMEs and calculation of the yield based on the average response factor of two internal standards (C13:0 and C23:0).

^c.^ FAME quantification based on response factors for individual fatty acids in an external FAME mix (C4-C24) normalized to the detected amount of IS (C13:0) in the sample.

Gravimetric yields, often referred to as total fat, varied from 44.7 to 16.2% (g/g biomass) in *M*.*circinelloides* and from 48.6 to 8.4% (g/g biomass) in *M*.*alpina*. The gravimetric yields exceeded the FAME yields by far. Although gravimetric lipid quantification in the Folch and Bligh methods are commonly used to evaluate the production of SCOs in microorganisms, it has been reported that gravimetric approaches overestimate the yield of fatty acids [35, 37]. Regardless of whether the SCO will be used for production of nutraceuticals, food, feed or biodiesel, the industrially important components are the fatty acids. Since the lipid extract contains other components than fatty acids that contribute to a higher weight, such as pigments, sterols, waxes and proteins or carbohydrates bound to lipids, gravimetric quantifications in general overestimate the potential of the microorganism as a SCO producer [35, 70]. Conversion of lipids into FAMEs by transesterification, followed by quantification in GC-FID provides a more reliable estimation of the single cell oil yield because it is a measure of fatty acids rather than total lipids. This becomes apparent when we compare the gravimetric yields after Lewis direct transesterification (44.7% in *M*.*circinelloides* and 48.6% in *M*.*alpina*), where the fatty acids in the lipid extract have already been converted into FAMEs, with the FAME yield determined in GC-FID for the same extract (25.8% in *M*.*circinelloides* and 29.0% in *M*.*alpina*). This shows that only 58 and 60% of the gravimetric lipid weight can be accounted for as FAMEs in *M*.*circinelloides* and *M*.*alpina*, respectively. Previous studies have shown that different lipid classes are converted into FAMEs to varying degrees, meaning that the composition of the SCO will affect the FAME yield [37, 71]. For instance, on a gravimetric basis 100% of triglycerides (TAGs) are converted into FAMEs while only 64% of phospholipids, such as phosphatidylcholine, are converted [37]. However, a TLC analysis of the extracted lipid classes for the different extraction methods showed that 80.9% and 95.1% of the lipids from *M*.*circinelloides* and *M*.*alpina* was converted into FAMEs through the Lewis transesterification method, respectively (Table B in S1 Table). The extracted lipid classes also varies slightly between Folch and Bligh extraction, but the major lipid class for both M.circinelloides and M.alpina is TAGs, which constitutes between 70–80% of the extracted lipids. This means that the variance between the gravimetric yield and the FAME yield observed in the present and previous studies cannot be explained by lipid class differences in FAME conversion. Thus, the higher gravimetric yield must be explained by other components in the lipid extract, like pigments, sterols, waxes and proteins or carbohydrates bound to lipids.

Addition of two internal standards (ISs), to cover the range of fatty acids present in the sample, can prevent the over- or underestimation of individual fatty acids that differ in chain length and unsaturation from a single internal standard [72]. Normally, heptadecanoic acid (C17:0) or nonadecanoic acid (C19:0) are preferred as internal standards. However, small amounts of C17:0 was found in *M*.*circinelloides*, while C19:0 elutes simultaneously with C18:3 in some columns, including the column used in this study, and hence, using them for quantification would overestimate the fatty acid content in these species. Thus, in our study, we evaluated the use of C13:0 and C23:0 as internal standards for quantification in GC-FID, in addition to external standard calibration (using a FAME mix) normalized to the amount of internal standard (C13:0) in the sample. The results showed similar lipid yields irrespective of whether the quantification had been performed using an averaged response factor derived from the two internal standards (C13:0 and C23:0) or by external calibration normalized to the amount of internal standard (C13:0) in the sample (Table 2). We hereby conclude that FAME quantification in GC-FID is the best-suited method for determining the lipid yield and profile in SCO producers, due to overestimation of the fatty acid yield by gravimetric approaches. We could not detect major differences between quantification using internal standards or external calibration. Hence, advantages and disadvantages of both quantification techniques needs to be taken into consideration. External calibration in GC-FID is more expensive and time-consuming, but the main advantages are the calculation of separate response factors for each individual fatty acid in the sample and the correction of potential losses during extraction accounted for by the normalization to the internal standard. Using internal standard quantification is less cost- and time-consuming, but careful consideration needs to be taken to select a suitable internal standard.

Fatty acid profiles, determined after transesterification and detection by GC-FID, were found to be similar irrespective of the extraction method in *M*.*circinelloides* and similar for Lewis and Folch extraction in *M*.*alpina*. (Table C in S1 Table). Fatty acids with a chain length longer than 20 carbons, except C20:4, could not be detected using the standard Bligh method in *M*.*alpina*. However, by increasing the solvent/sample ratio in the Bligh method, a fatty acid profile similar to that achieved by Lewis and Folch extraction in *M*.*alpina* could be obtained.

## Conclusions

Lipid extraction is a crucial step in the screening of oleaginous microorganisms and the following optimization of SCO production. Thus, the selection of a suitable extraction method is important in order to perform an adequate evaluation of the lipid yield and profile. Lipid losses can occur for any extraction method applied for microbial biomass. This study has shown that, depending on the extraction method and microbial biomass employed, significant variations in lipid yield can be obtained. Thus, it is difficult to appoint a single extraction method as a golden standard, especially when extraction methods are applied to microorganisms that have not been extensively studied previously. FTIR spectroscopy is commonly used in microbial research for the identification and characterization of microorganisms, but this study has shown that FTIR spectroscopy applied to intact biomass and biomass residual after lipid extraction can serve as a tool for evaluating the lipid extraction efficiency. The presence or absence of lipid bands in the IR spectrum of the biomass after extraction can function as a measure for the efficiency of the extraction method. Since an FTIR spectrum represents an overall biochemical fingerprint, it can be used in parallel to identify components that may affect lipid extraction processes, for example chitin, glucuronans and polyphosphates in fungal biomass.

## Supporting Information

S1 Text Cultivation conditions. Information on maintenance and cultivation of the fungal strains Mucor circinelloides and Mortierella alpina for lipid production.
(PDF)

S1 Fig FTIR spectra. FTIR spectra of glucuronic acid and glycerol standards (Fig A). Spectra of *M*.*alpina* and *M*.*circinelloides* biomass grown under non-lipid producing conditions (Fig B). Infrared spectra of fungal biomass before and after lipid extraction when a modified Bligh method was applied (Fig C). FTIR spectra of acid hydrolysis treatment of *M*.*alpina* biomass (Fig D). Different bead beating exposure times for Folch, Bligh and Lewis extraction from *M*.*circinelloides* biomass (Fig E).
(PDF)

S1 Table Bead beating FAME yield, lipid class and fatty acid profiles. Effect of bead beating exposure time on FAME yield (Table A). TLC analysis of lipid classes after Bligh, Folch and Lewis extractions (Table B). Fatty acid profiles after different extraction methods (Table C).
(PDF)

S1 Dataset Raw FTIR spectral data. (XLSX)
