# Supplementary material for: FTIR Spectroscopy for Evaluation and Monitoring of Lipid Extraction Efficiency for Oleaginous Fungi
Source: PLoS One. 2017 Jan 24;12(1):e0170611. doi: 10.1371/journal.pone.0170611 (PMC5261814; doi:10.1371/journal.pone.0170611)
Supplement: S1 Text — Information on maintenance and cultivation of the fungal strains Mucor circinelloides and Mortierella alpina for lipid production. (PDF) [file pone.0170611.s001.pdf]

S1 Text Cultivation Conditions

Information on maintenance and cultivation of the fungal strains *Mucor circinelloides* and *Mortierella alpina* for lipid production.

FTIR Spectroscopy for Evaluation and Monitoring of Lipid Extraction Efficiency for Oleaginous Fungi

Kristin Forfang[1, 2*], Boris Zimmermann[1], Gergely Kosa[1,2], Achim Kohler[1], Volha Shapaval[1,2].

[1]Department of Mathematical Sciences and Technology, Norwegian University of Life Sciences, Drøbakveien 31, Ås, Norway.
[2]Nofima AS, Osloveien 1, 1430 Ås, Norway.

*Corresponding author:

Kristin Forfang
Department of Mathematical Sciences and Technology
Norwegian University of Life Sciences
Drøbakveien 31, 1430 Ås
Tel: +47 672 31 643
E-mail: kristin.forfang@nmbu.no

Table of Contents

Cultivation conditions…………………………………………………………… S1 Text-2

Cultivation Conditions

Mortierella alpina was maintained on potato dextrose agar (PDA, VWR Chemicals Prolabo, Leuven) at 4 °C with sub culturing every two months (7 days, 28 °C). Inoculum of *M.alpina* was prepared by suspending the mycelia from one freshly grown agar plate in physiological salt solution (0.9 % NaCl). Spores of *Mucor circinelloides* were maintained in glycerol stocks at -80 °C. Fresh spore suspension of *M.circinelloides* was prepared by inoculating malt extract agar (MEA, Merck, Germany) with 3 x 10 µL of the glycerol stock, incubating at 28 °C for 7 days and suspending the spores in 10 mL physiological salt solution.

Fungal strains were cultured in a lipid-producing medium containing (gL^{-1}): glucose 80, yeast extract 3, $MgSO_4 \cdot 7H_2O$ 1.88, KH_2PO_4 8.75, Na_2HPO_4 1.25, $CaCl_2 \cdot 2H_2O$ 0.12 with the addition of 1000X Trace Element Solution (TES: $CoSO_4 \cdot 7H_2O$ 0.1, $CuSO_4 \cdot 5H_2O$ 0.1, $FeCl_3 \cdot 6H_2O$ 8.0, $MnSO_4 \cdot 7H_2O$ 0.01, $ZnSO_4 \cdot 7H_2O$ 1.0). Fungal strains were also cultivated in non-lipid producing media, malt extract broth for *M.circinelloides* (MEB, Merck Millipore, Germany) and potato extract broth for *M.alpina* (Sigma-Aldrich, USA). Cultivations were carried out in 1L shake flasks containing 200 mL medium at 28°C with 120 rpm shaking. Media was inoculated with 200 µL spore suspension of *M.circinelloides* (2×10^5 spores/mL, final) and 1 mL mycelium suspension of *M.alpina*.
