# Supplementary material for: FTIR Spectroscopy for Evaluation and Monitoring of Lipid Extraction Efficiency for Oleaginous Fungi
Source: PLoS One. 2017 Jan 24;12(1):e0170611. doi: 10.1371/journal.pone.0170611 (PMC5261814; doi:10.1371/journal.pone.0170611)
Supplement: S1 Fig — FTIR spectra of glucuronic acid and glycerol standards (Fig A). Spectra of M.alpina and M.circinelloides biomass grown under non-lipid producing conditions (Fig B). Infrared spectra of fungal biomass before and after lipid extraction when a modified Bligh method was applied (Fig C). FTIR spectra of acid hydrolysis treatment of M.alpina biomass (Fig D). Different bead beating exposure times for Folch, Bligh and Lewis extraction from M.circinelloides biomass (Fig E). (PDF) [file pone.0170611.s002.pdf]

S1 Fig FTIR spectra

This supporting information includes: FTIR spectra of glucuronic acid and glycerol standards (Fig A); Spectra of *M.alpina* and *M.circinelloides* biomass grown under non-lipid producing conditions (Fig B); Infrared spectra of fungal biomass before and after lipid extraction when a modified Bligh method was applied (Fig C); FTIR spectra of acid hydrolysis treatment of *M.alpina* biomass (Fig D); Different bead beating exposure times for Folch, Bligh and Lewis extraction from *M.circinelloides* biomass (Fig E).

FTIR Spectroscopy for Evaluation and Monitoring of Lipid Extraction Efficiency for Oleaginous Fungi

Kristin Forfang[1, 2*], Boris Zimmermann[1], Gergely Kosa[1,2], Achim Kohler[1], Volha Shapaval[1,2].

[1]Department of Mathematical Sciences and Technology, Norwegian University of Life Sciences, Drøbakveien 31, Ås, Norway.
[2]Nofima AS, Osloveien 1, 1430 Ås, Norway.

*Corresponding author:

Kristin Forfang
Department of Mathematical Sciences and Technology
Norwegian University of Life Sciences
Drøbakveien 31, 1430 Ås
Tel: +47 672 31 643
E-mail: kristin.forfang@nmbu.no

Table of Contents

FTIR spectra of glucuronic acid and glycerol.. S1 Fig-2
Spectra of *M.alpina* and *M.circinelloides* biomass grown
under non-lipid producing conditions... S1 Fig-3
Modification of the Bligh extraction method.. S1 Fig-4
Acid hydrolysis pretreatment of *M.alpina* biomass.................................. S1 Fig-5
Bead beating exposure time... S1 Fig-6

FTIR spectra of glucuronic acid and glycerol

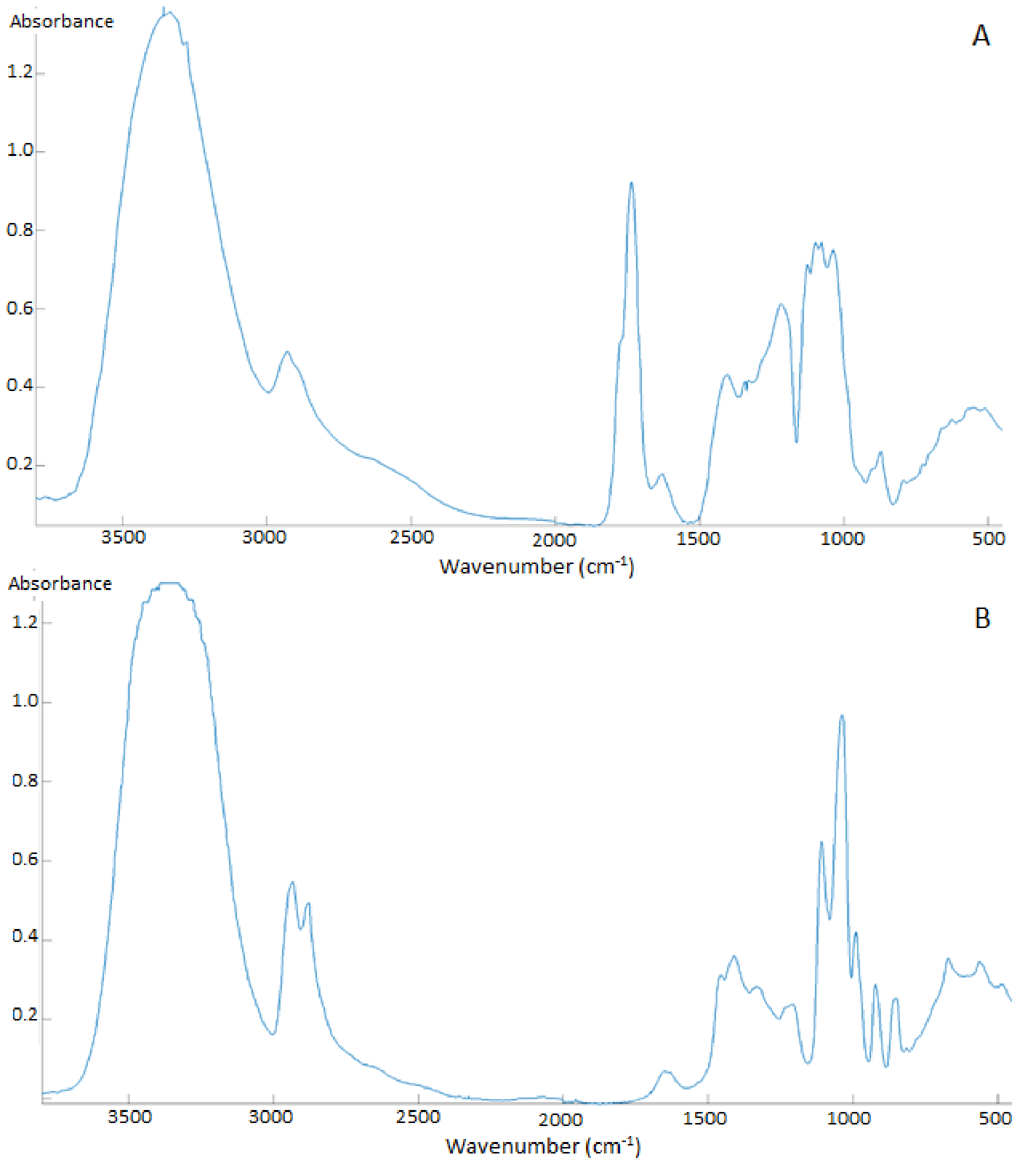

Fig A. FTIR spectra of glucuronic acid and glycerol. Infrared spectra were recorded of standard compounds glucuronic acid (A) and glycerol (B).

Spectra of *M.alpina* and *M.circinelloides* biomass grown under non-lipid producing conditions

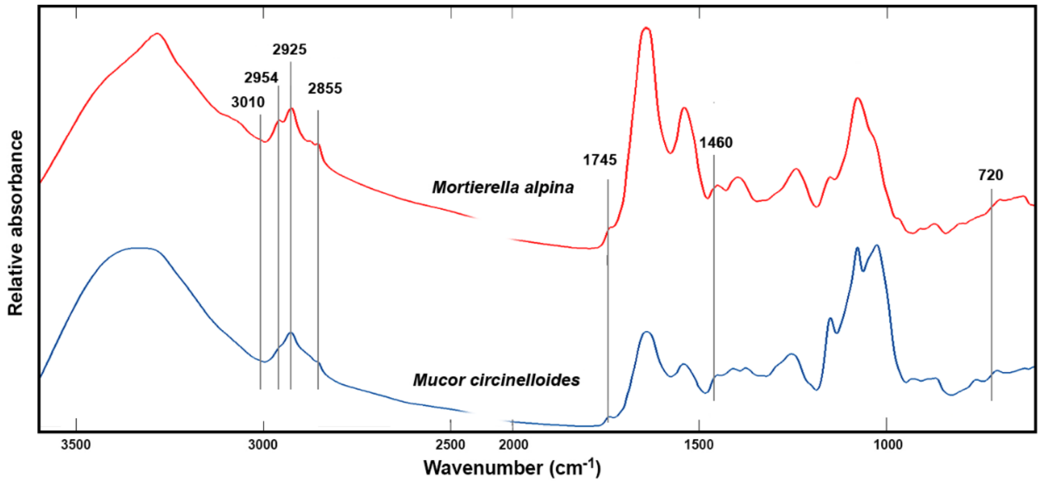

Fig B. Spectra of non-lipid producing fungal biomass. FTIR spectra of *Mortierella alpina* and *Mucor circinelloides* biomass grown under non-lipid producing conditions (no nitrogen limitation).

Modification of the Bligh extraction method

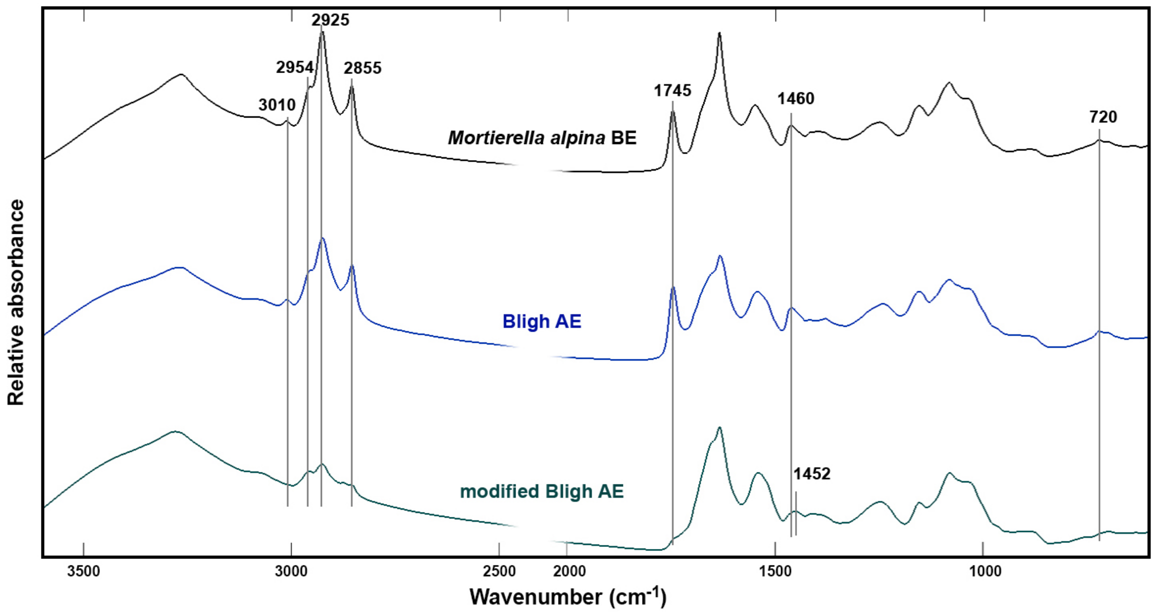

Fig C. Modification of the Bligh method. Infrared spectra of *Mortierella alpina* biomass were recorded before (BE) and after extraction (AE) using the original Bligh method and a modified Bligh method (Bligh 20:1). In the modified method, the initial solvent/sample ratio was increased from 3:1 to 20:1 (similar to the ratio applied in the Folch method).

Acid hydrolysis pretreatment of *M.alpina* biomass

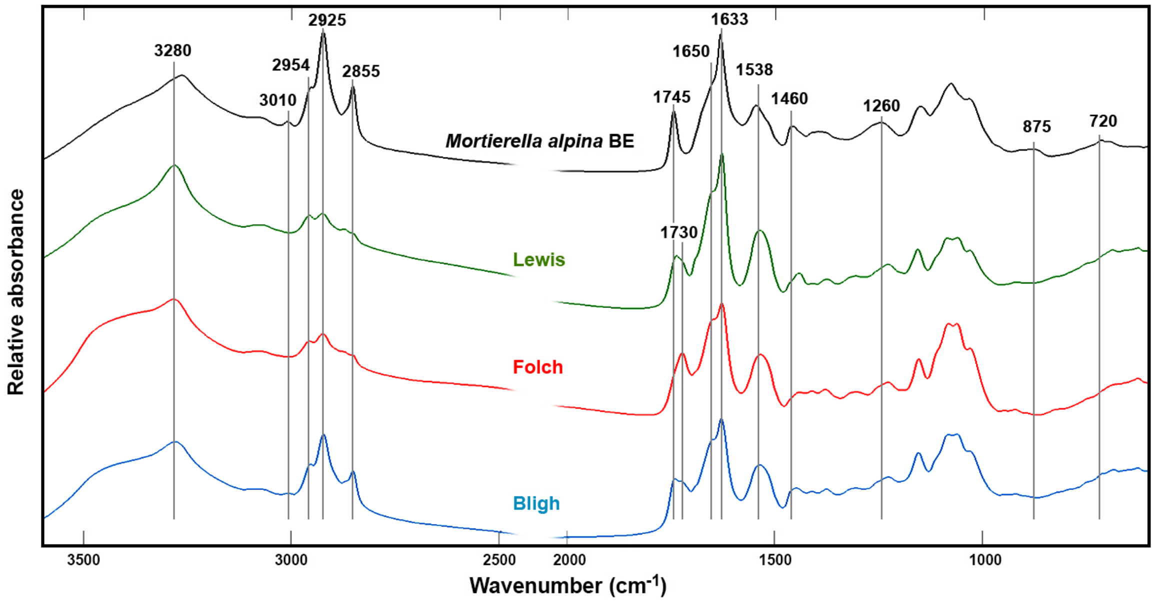

Fig D. Acid hydrolysis pretreatment of *M.alpina* biomass. Infrared spectra of *Mortierella alpina* biomass were recorded before (BE) and after acid hydrolysis pretreatment and extraction using the methods of Bligh, Folch and Lewis.

Bead beating exposure time

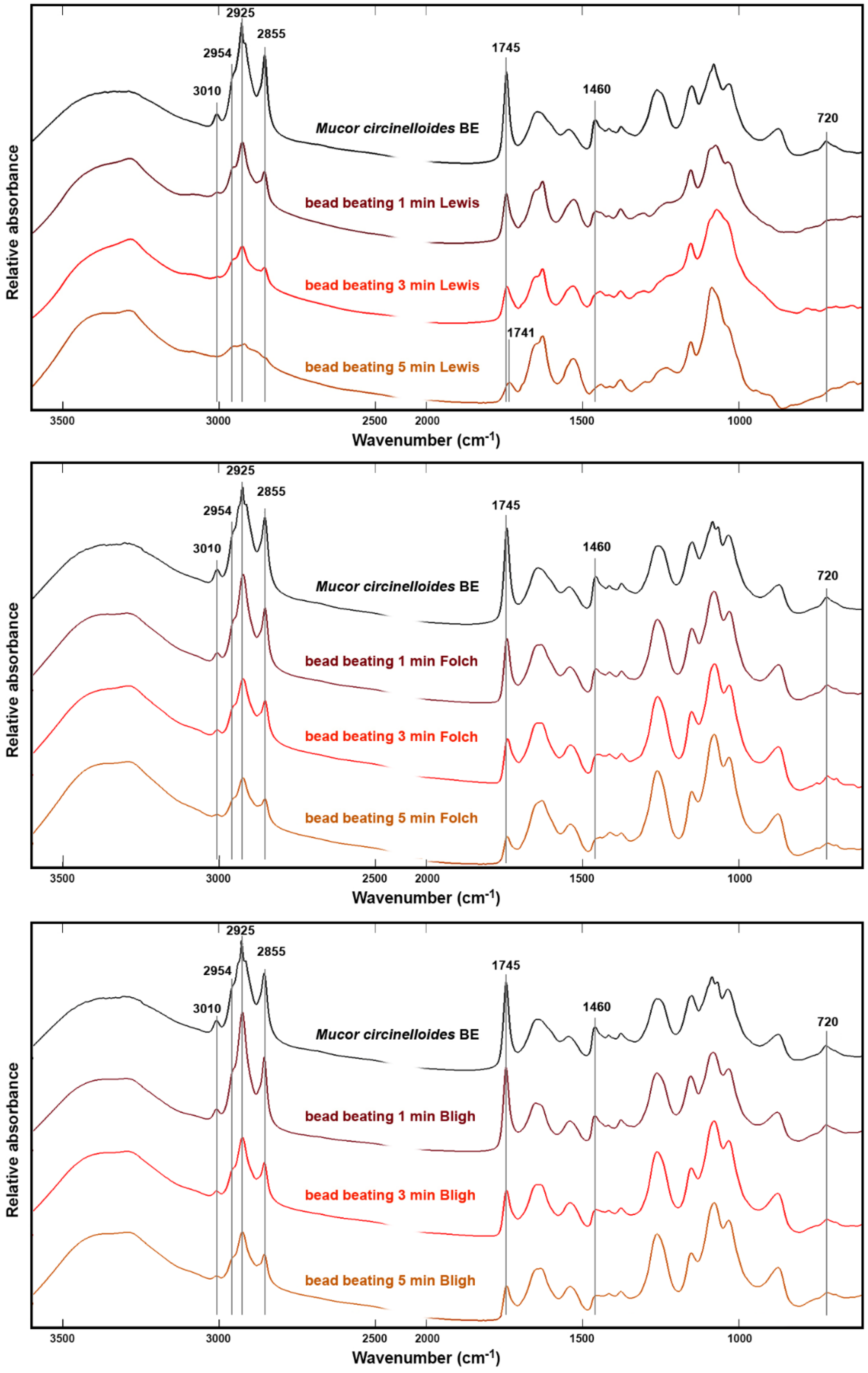

Fig E. FTIR spectra of *M.circinelloides* biomass after bead beating exposure. *Mucor circinelloides* biomass was disrupted by bead beating for 1, 3 and 5 min and infrared spectra were recorded of the biomass before (BE) and after extraction using the Bligh, Folch and Lewis methods.
