# Supplementary material for: FTIR Spectroscopy for Evaluation and Monitoring of Lipid Extraction Efficiency for Oleaginous Fungi
Source: PLoS One. 2017 Jan 24;12(1):e0170611. doi: 10.1371/journal.pone.0170611 (PMC5261814; doi:10.1371/journal.pone.0170611)
Supplement: S1 Table — Effect of bead beating exposure time on FAME yield (Table A). TLC analysis of lipid classes after Bligh, Folch and Lewis extractions (Table B). Fatty acid profiles after different extraction methods (Table C). (PDF) [file pone.0170611.s003.pdf]

S1 Table Bead Beating FAME Yield, Lipid Class and Fatty Acid Profiles

This supporting information includes the following: Effect of bead beating exposure time on FAME yield (Table A); Lipid classes after Bligh, Folch and Lewis extractions (Table B); Fatty acid profiles after different extraction methods (Table C).

FTIR Spectroscopy for Evaluation and Monitoring of Lipid Extraction Efficiency for Oleaginous Fungi

Kristin Forfang[1, 2*], Boris Zimmermann[1], Gergely Kosa[1,2], Achim Kohler[1], Volha Shapaval[1,2].

[1]Department of Mathematical Sciences and Technology, Norwegian University of Life Sciences, Drøbakveien 31, Ås, Norway.
[2]Nofima AS, Osloveien 1, 1430 Ås, Norway.

*Corresponding author:

Kristin Forfang
Department of Mathematical Sciences and Technology
Norwegian University of Life Sciences
Drøbakveien 31, 1430 Ås
Tel: +47 672 31 643
E-mail: kristin.forfang@nmbu.no

Table of Contents

Table A. Effect of bead beating exposure time on lipid extraction yield from M.circinelloides……………………………………………………….. S1 Table-2
Table B. TLC analysis of lipid classes after Bligh, Folch and Lewis extraction……………………………………………………………… S1 Table-2
Table C. Fatty acid profiles for *M.circinelloides* and *M.alpina*……………….. S1 Table-3

Table A. Effect of bead beating exposure time on lipid extraction yield from *M.circinelloides* biomass.

FAME yield (mg/g biomass)				
**Extraction method**	Bead beating (min)			
	0	1	3	5
Bligh	128.3 ± 34.8	104.5 ± 13.7	181.2	185.9
Folch	113.0 ± 13.2	253.3 ± 14.8	219.6	272.0
Lewis	258.4 ± 3.8	313.5 ± 17.5	511.7	623.4

Table B. TLC analysis of extracts after lipid extraction from *Mucor circinelloides* and *Mortierella alpina* biomass using the methods of Bligh, Folch and Lewis. The composition of triacylglycerols (TAG), free fatty acids (FFA), mono- and diacylglycerols (MDGs), phospholipids (PL) and fatty acid methyl esters (FAME) are given in percentages.

Lipid class composition (%)						
**Strain**	Extraction method	TAG	FFA	MDGs	PL	FAME
M.circinelloides	Bligh	69,9	10,7	10,3	9,2	0,0
	Folch	73,1	16,9	5,1	5,0	0,0
	Lewis	7,21	9,56	1,08	1,20	80,9
M.alpina	Bligh	74,3	14,3	6,9	4,5	0,0
	Folch	77,5	16,3	3,0	3,2	0,0
	Lewis	0,0	0,0	2,4	2,5	95,1

Table C. Fatty acid profiles for *Mucor circinelloides* and *Mortierella alpina* after lipid extraction using the methods of Bligh, Folch and Lewis.

FAME composition (% of total FAME)							
**Fatty acid**	*M. circinelloides*			*M. alpina*			
	Bligh	Folch	Lewis	Bligh	Folch	Lewis	Mod. Bligh[a]
C14:0	1.65 ± 0.05	1.85 ± 0.04	1.99 ± 0.03		2.18 ± 0.04	2.09 ± 0.02	1.64
C15:0		0.71 ± 0.02					
C16:0	16.51 ± 0.01	15.99 ± 0.04	16.71 ± 0.02	24.83 ± 2.43	20.09 ± 0.07	20.22 ± 0.03	18.55
C16:1	5.17 ± 0.10	5.07 ± 0.05	5.57 ± 0.05				
C17:0	1.65 ± 0.01	2.16 ± 0.00	2.00 ± 0.02				
C17:1	0.95 ± 0.01	1.19 ± 0.00	1.13 ± 0.01				
C18:0	4.28 ± 0.08	4.47 ± 0.04	4.45 ± 0.02	11.76 ± 2.58	8.70 ± 0.36	8.66 ± 0.03	7.59
C18:1	36.80 ± 0.59	26.86 ± 0.04	28.9 ± 0.06	29.80 ± 1.80	23.17 ± 0.16	23.48 ± 0.06	27.50
C18:2	21.42 ± 0.39	26.19 ± 0.01	25.86 ± 0.00	19.47 ± 0.70	14.99 ± 0.37	15.33 ± 0.03	11.21
C18:3	11.56 ± 0.26	13.77 ± 0.04	13.37 ± 0.00	5.40 ± 7.64	7.34 ± 0.46	7.56 ± 0.00	5.86
C20:0					0.80 ± 0.05	0.76 ± 0.00	0.63
C20:1					1.20 ± 0.05	1.22 ± 0.00	0.00
C22:0					6.00 ± 0.36	5.01 ± 0.02	1.61
C20:3		1.74 ± 0.18			5.20 ± 0.07	5.37 ± 0.01	3.93
C20:4				8.74 ± 0.13	8.10 ± 0.08	8.33 ± 0.06	18.59
C24:0					2.22 ± 0.15	1.97 ± 0.01	1.86

[a.] Initial solvent/sample ratio was increased from 3:1 to 20:1
